# Corticomuscular Transmission of Tremor Signals by Propriospinal Neurons in Parkinson's Disease

**DOI:** 10.1371/journal.pone.0079829

**Published:** 2013-11-20

**Authors:** Manzhao Hao, Xin He, Qin Xiao, Bror Alstermark, Ning Lan

**Affiliations:** 1 Institute of Rehabilitation Engineering, Med-X Research Institute, School of Biomedical Engineering, Shanghai Jiao Tong University, Shanghai, China; 2 Department of Neurology and Institute of Neurology, Ruijin Hospital affiliated to Shanghai Jiao Tong University School of Medicine, Shanghai, China; 3 Department of Integrative Medical Biology, Umea University, Umea, Sweden; 4 Division of Biokinesiology and Physical Therapy, University of Southern California, Los Angeles, California, United States of America; Georgia State University, United States of America

## Abstract

Cortical oscillatory signals of single and double tremor frequencies act together to cause tremor in the peripheral limbs of patients with Parkinson's disease (PD). But the corticospinal pathway that transmits the tremor signals has not been clarified, and how alternating bursts of antagonistic muscle activations are generated from the cortical oscillatory signals is not well understood. This paper investigates the plausible role of propriospinal neurons (PN) in C3–C4 in transmitting the cortical oscillatory signals to peripheral muscles. Kinematics data and surface electromyogram (EMG) of tremor in forearm were collected from PD patients. A PN network model was constructed based on known neurophysiological connections of PN. The cortical efferent signal of double tremor frequencies were integrated at the PN network, whose outputs drove the muscles of a virtual arm (VA) model to simulate tremor behaviors. The cortical efferent signal of single tremor frequency actuated muscle spindles. By comparing tremor data of PD patients and the results of model simulation, we examined two hypotheses regarding the corticospinal transmission of oscillatory signals in Parkinsonian tremor. Hypothesis I stated that the oscillatory cortical signals were transmitted via the mono-synaptic corticospinal pathways bypassing the PN network. The alternative hypothesis II stated that they were transmitted by way of PN multi-synaptic corticospinal pathway. Simulations indicated that without the PN network, the alternating burst patterns of antagonistic muscle EMGs could not be reliably generated, rejecting the first hypothesis. However, with the PN network, the alternating burst patterns of antagonist EMGs were naturally reproduced under all conditions of cortical oscillations. The results suggest that cortical commands of single and double tremor frequencies are further processed at PN to compute the alternating burst patterns in flexor and extensor muscles, and the neuromuscular dynamics demonstrated a frequency dependent damping on tremor, which may prevent tremor above 8 *Hz* to occur.

## Introduction

Resting tremor in Parkinson's disease (PD) is elicited by the reciprocal and alternating activities of antagonistic muscles [1]. The tremor of variable frequency between 3 and 7 *Hz* is originated from oscillatory neuronal activities in subcortical and cortical networks [2], [3]. Correlation studies have been performed between electroencephalography (EEG) and electromyography (EMG) [4], [5], magnetoencephalography (MEG) and EMG [6]–[8], and local field potential (LFP) in subthalamic nucleus and EMG [9]. All studies revealed that peripheral EMG showed strong coupling with neuronal oscillatory activity in the brain at single tremor frequency and double tremor frequency. It was believed that these two oscillation sources possibly involve different cerebral networks and different pathways to the periphery [5], [10]. A further analysis indicated that the cortical oscillatory activity at double tremor frequency was the main central drive contributing to corticomuscular coupling [11]. However, the spinal mechanism of corticomuscular processing remains unknown with regard to how the cerebral oscillations of single and double frequencies are transformed into alternating pattern of antagonistic muscle bursts that generate tremor behaviors.

There are two major descending pathways from primary motor cortex to spinal motor neurons, a mono-synaptic cortico-motoneuronal (CM) pathway, and a multi-synaptic cortico-motoneuronal pathway via interneurons [12]–[15]. The direct mono-synaptic pathway appears unique for higher primates [16]. For the multi-synaptic pathway, a special group of interneurons located at the C3–C4 levels have been revealed, i.e. the propriospinal neurons (PN) [17], [18]. The PN network has been identified and described by extensive physiological experiments in cats and macaque monkeys [19], [20], and implicated in human by electrophysiological investigations [15], [19]. The studies in cats and primates have shown that the PNs are subject to multiple excitatory and inhibitory influences by cortical inputs, and the PN network is involved in reaching movement control [20]. The specific roles of PN in human movement control have not been well understood because of limitations of experimental techniques to record the PN activities during task performance. Evidence has also been found that normal cyclic movements performed in human involved a spinal interneuronal network that processes the cortical motor commands [21], [22]. But the nature of the spinal interneuronal network was still unknown.

Observations of cortical signals and peripheral behaviors of PD tremor may provide the input-output information that allows us to investigate the corticospinal mechanism of tremor transmission [7], [8], [11], [23], [24]. Based on MEG and simultaneous EMG recordings [7], [23], Timmermann and colleagues assumed that a spinal circuitry was responsible to divide the primary motor cortex (M1) outputs into the bursting antagonistic activities [8], [11]. But it was not possible to pinpoint from these data the spinal mechanism of corticomuscular coupling with regard to how the cerebral oscillation signals of single and double tremor frequencies are transformed into the alternating pattern of antagonistic muscle bursts that generate the tremor behaviors. In this paper, we have taken a computational approach to address the issue of corticomuscular transmission in tremor generation. We hypothesized that the PN network in C3–C4 levels plays a pivotal role in translating cortical drives into alternating activities of flexor and extensor muscles. A PN network model was used to understand the premotorneuronal processing of tremor signals, which was based on neural connections identified in extensive neurophysiological studies [20], [25]. With the PN model, a computational model of the corticomuscular coupling was constructed to examine hypotheses regarding the involvement of PN in the generation of Parkinsonian tremor.

We also designed experiments to record resting tremor and EMGs signals from PD patients with their arm performing postural tasks in the horizontal plane with gravity-support cast on a frictionless glass surface [24]. The measurements of resting tremor and EMG patterns of antagonistic muscles in the horizontal plane were used to compare with the simulated tremor behaviors and muscle activation patterns. Model simulations were performed with the virtual arm positioned in the horizontal plane to generate resting tremor behaviors under cortical inputs with and without the PN network. Hypotheses were rejected or accepted based on the similarity between experimentally observed and simulated tremor behaviors. The results of comparison indicated that the PN network plays the key role in dividing the cortical oscillatory signals into alternating burst pattern of flexor/extensor muscle activations. Preliminary results have also been reported elsewhere in a conference proceeding [26].

## Materials and Methods

**Figure 1:**
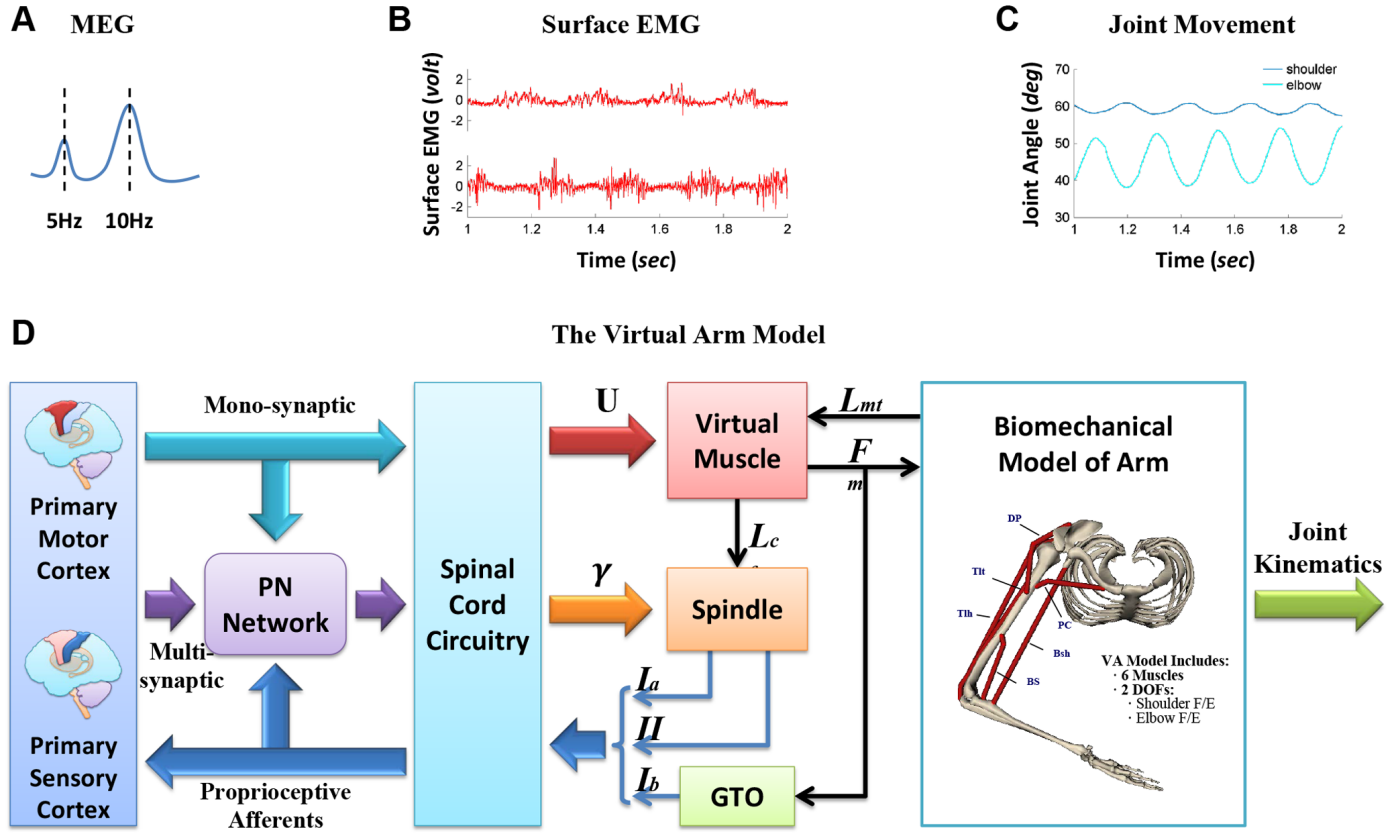
Experimental recordings of (A) cortical oscillations, (B) surface Electromyography (sEMG), (C) joint kinematics of tremor recorded from Parkinson's patient (PD), and (D) the Corticospinal Virtual Arm (CS-VA) model used in this study. (**A**) The frequency spectral analysis of Magnetoencephalography (MEG) [7] of Parkinson's patients exhibited abnormal synchronization of neural activities in the brain at the single tremor frequency (about 5 *Hz*) and the double tremor frequency (about 10 *Hz*), which were identified as the central driving signals for the Parkinsonian tremor. Thus in this study, 5 *Hz* and 10 *Hz* oscillatory activities from the primary motor cortex were assumed as CS-VA model inputs to elicit tremor behaviors in the model. (**B**) Alternating burst patterns were observed from EMGs of a pair of antagonists (e.g. Biceps and Triceps), which corresponded to the 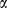-activation patterns of the Virtual Muscle model. (**C**) Rhythmic joint movements of Parkinson tremor observed in PD patients were used as the criteria to assess whether the model outcome of simulation reproduced the PD tremor. (**D**) The CS-VA model developed in this study consisted of the corticospinal pathways and the peripheral sensorimotor virtual arm (VA). The two descending pathways were mono-synaptic pathway from the primary motor cortex to the motoneuron pool and multi-synaptic pathway mediated by the propriospinal neuronal (PN) network, and the ascending pathway of proprioceptive afferents to the primary sensory cortex. The sensorimotor VA model included spinal cord circuitry, virtual muscle, proprioceptors (muscle spindle and Golgi tendon organ, GTO), and musculoskeletal dynamics, was validated to capture the realistic properties of human upper extremity [24]. The virtual arm has two degree of freedoms (DOFs) with three pairs of antagonistic muscles: Pectoralis Major Clavicle (PC) and Deltoid Posterior (DP) for shoulder, Brachialis (BS) and Triceps Lateral (Tlt) for elbow; Biceps Short Head (Bsh) and Triceps Long Head (Tlh) across both joints.

In this study, a model was developed to simulate tremor behaviors driven by cortical oscillatory inputs (Figure 1 and sections 2.2, 2.3). Simulations were performed with two different descending pathways: the mono-synaptic cortico-motoneuronal pathway and the multi-synaptic PN pathway, respectively. Kinematics data of tremor and EMG data were recorded from PD patients, and were used as a template to compare with those of simulated data.

### Recordings in PD Subjects and Ethics Statement

Seven patients (four females, three males, ages 62±3.16 years, height 164±7.21 cm, weight 64.67±9.78 kg) with idiopathic tremor-dominant Parkinson's disease were recruited for this study. All subjects had no other neurogenic disease. The study was approved by the Ethics Committee of Animal and Human Subject Studies of Shanghai Jiao Tong University. All subjects signed the informed consent form, and were instructed by the attending physician to take their prescribed medications as usual while participating the experiment session.

An experimental method was designed to record resting tremor in PD patients without the effect of gravity [24]. For clarity, the experiment procedure was described in this paper in the following. During data collection, the subjects were seated comfortably in a chair at a table with adjusted height, instructed to perform posture maintenance tasks in the horizontal plane, using upper extremity on the tremor-affected side. A fiberglass brace apparatus was worn on the forearm of the subject, which was supported by a ball-bearing base against gravity. The ball-bearing base could move on a frictionless glass surface to allow the resting tremor to occur freely at the shoulder and elbow joints. The wrist was loosely fixed, and the tremor at the wrist was not recorded, because our model could not simulate wrist tremor. Kinematics and surface electromyography (EMG) data were collected employing a MotionMonitor II system (Inn Sports Inc., Chicago, IL, USA), which was particularly suitable for movement monitoring in the upper extremity and EMG signal recording. We collected the surface EMG of two pairs of antagonists: biceps brachii (Biceps), triceps brachii (Triceps), flexor digitorum superficialis (FDS), and extensor digitorum (ED), while PD subjects performed posture tasks. Subjects were asked to maintain their hand pointer on the brace at positions in the horizontal plane indicated by dark spots on the glass surface. Subjects performed postural tasks with resting tremor, during which tremor and EMGs were recorded for 15 *seconds* in each trial. The task was repeated 10 times at each posture for each subject. The movement was captured using the MotionMonitor II system with a 120 frame per second resolution. Surface EMG signals were amplified with a gain of 5000, then band-pass filtered with 1 *Hz* high-pass and 1000 *Hz* low-pass filter, and then sampled at 2410 *Hz*. The spectrum of sampled EMG and motion data were calculated using FFT algorithm in MATLAB (Mathworks, Natick, MA, USA). The temporal patterns of tremor kinematics and RMS EMG were used later for comparison with simulated tremor behaviors.

### The Corticospinal-Virtual Arm (CS-VA) Model

The corticospinal-virtual arm (CS-VA) model consisted of a propriospinal neuron (PN) network [20] (see Section 2.3), a spinal reflex (SR) circuitry [27]–[30] and the virtual arm (VA) model [30]–[35] (shown in Figure 1). The subsystems of SR and VA models were based on physiological studies and examined in previous computational studies of the spinal reflexes and neuromechanical behaviors [30]–[38]. These component models were integrated in the SIMULINK/MATLAB (version 2010a) platform for simulation. The neuronal signal flow in the CS-VA model was also displayed in Figure 1. The inputs to the CS-VA model were cortical oscillation signals of single tremor frequency (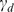) and double tremor frequency (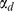), which were integrated at the PN via inhibitory and excitatory connections. The signal of single tremor frequency actuated the spinal gamma motoneurons (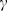-MNs) directly. The outputs of PNs were the main input signals to the spinal alpha motoneurons (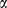-MNs), which was regulated by spinal reflexes of *Ia* and *Ib* afferents of muscle spindles and Golgi Tendon Organ (GTO), as well as recurrent inhibition of Renshaw cells. The outputs of 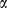-MNs activated the virtual muscles (VM) in the VA model to produce tremor behaviors.

At the bottom of the system, the generic VA model included components of virtual muscle (VM), biomechanical model of musculoskeletal dynamics, proprioceptor models (muscle spindle and GTO), and spinal reflex circuitry. The VA model was validated to capture the realistic neurophysiological and neuromechanical characteristics of the sensorimotor system of human upper extremity [30], [34], [37]. The VA model had two degrees of freedom in horizontal plane (shoulder flexion/extension, elbow flexion/extension), and was controlled by three pairs of antagonistic muscles. Two pairs were mono-articular muscles for each joint: Pectoralis Major Clavicle (PC) and Deltoid Posterior (DP) for shoulder, Brachialis (BS) and Triceps Lateral (Tlt) for elbow; and one pair was bi-articular muscle across both joints: Biceps Short Head (Bsh) and Triceps Long Head (Tlh).

The spinal reflex circuitry regulated the outputs of 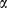-MNs according to afferents provided by *Ia*, *Ib* and recurrent feedback information. It was shown that the values of reflex gains must be kept relatively low in order to maintain the VA in a stable state, except for *Ib* afferent [30], [38]. This was due to the time delay in neural transmission from the peripheral sensory organs back to the spinal 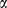-MN pools. Thus in this study, we have fixed the values of reflex gains in their stable range. This would make sure that oscillatory behaviors generated in simulations with this CS-VA model were caused by the cortical oscillatory inputs. The values of reflex gains used in this study were tabulated in Table 1.

**Table 1:** Parameter Settings of CS-VA Model Simulations.

	PN^1^ Gains			Reflex Gains					
Muscles	a^2^	d^3^	p^4^	r^5^	s^6^	b^7^	g^8^	*α_s_*	*γ_s_*
**PC** 9	0.1	1	0	0.1	0.2	0.1	0.2	0.1250	0.6335
**DP** 10	0.1	1	0	0.1	0.2	0.1	0.2	0.1375	0.5545
**Bsh** 1	0.1	1	0	0.1	0.2	0.1	0.2	0.1125	0.5618
**Tlh** 11	0.1	1	0	0.1	0.2	0.1	0.2	0.1375	0.5164
**BS** 12	0.1	1	0	0.1	0.2	0.1	0.2	0.1050	0.6095
**Tlt** 13	0.1	1	0	0.1	0.2	0.1	0.2	0.0750	0.5252

1 Propriospinal Neurons.

2 gains of *Ia* afferent to PN.

3 gains of descending gamma command.

4 gains of PN to reciprocal inhibition.

5 Reciprocal inhibition gains.

6 Stretch reflex gains.

7 Golgi Tendon Organ reflex gains.

8 Recurrent inhibition gains.

9 Pectoralis major Clavicle portion.

10 Deltoid Posterior.

11 Triceps long head.

12 Brachialis.

13 Triceps lateral head.

### The C3-C4 Propriospinal Neuron (PN) Model

**Figure 2:**
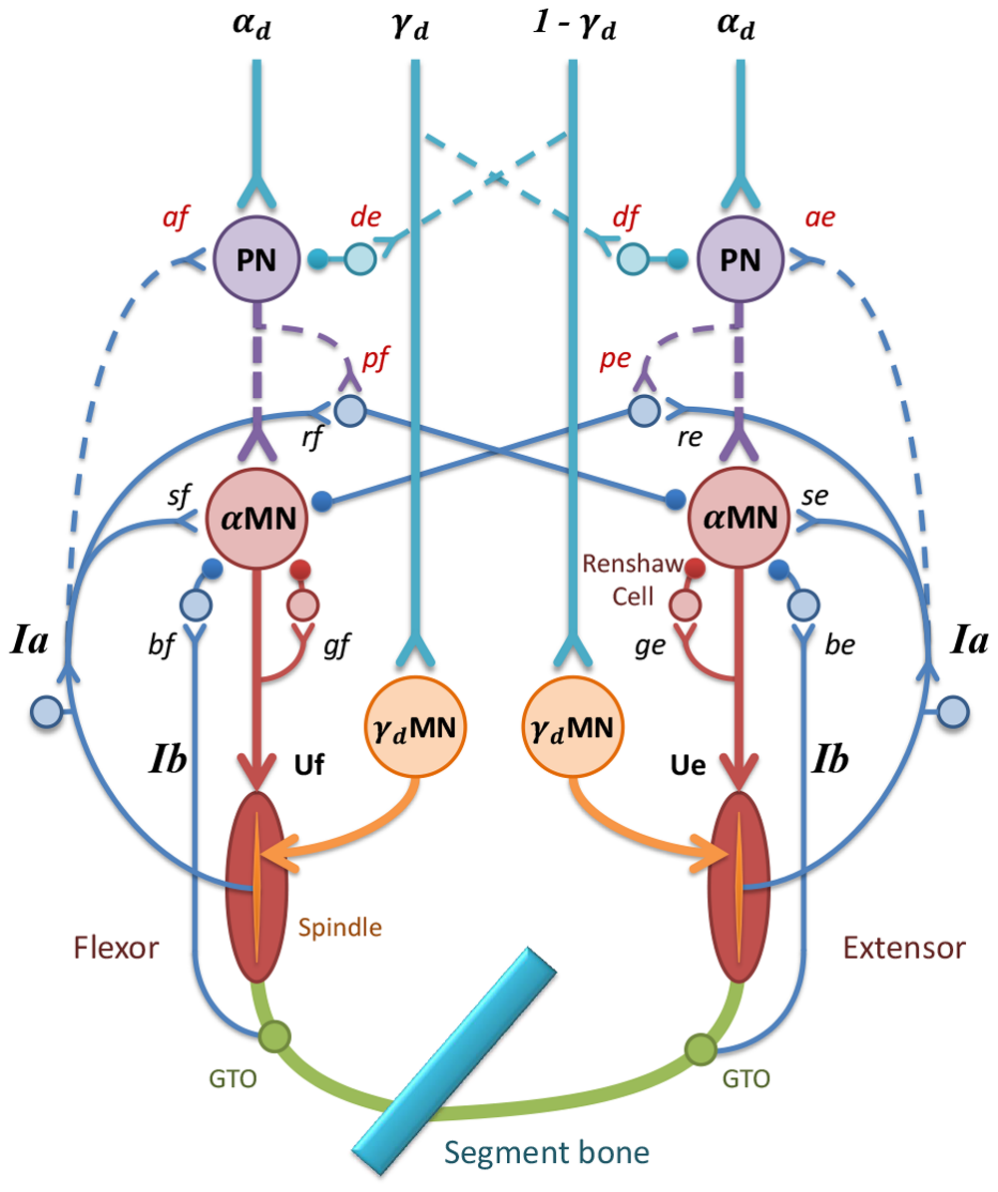
The model of Propriospinal Neuronal (PN) network in the corticospinal pathways of one pair of antagonistic muscles is illustrated here. This model was built based on experimentally identified PN connections (in dash lines) and spinal reflex circuitry. Subscript “*d*” of 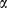 and 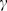 descending commands refers to “dynamic”. “*f*” refers to “flexor” and “*e*” refers to “extensor”. d_e_ and d_f_ are the inhibition gains of 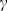 descending commands to PNs. *a_f_* and *a_e_* are gains of *Ia* to PN. *p_f_* and *p_e_* are the PN related reciprocal gains. *s_f_* and *s_e_* are stretch reflex gains. *r_f_* and *r_e_* are *Ia*-reciprocal inhibition gains. *g_f_* and *g_e_* are Renshaw cell gains. *b_f_* and *b_e_* are GTO feedback gains. The outputs of the GTO and spindles are feedback to the spinal, and are integrated with the descending and PN-processed signals, to produce activating signals (U*_f_* and U*_e_*) to control the muscles.

The C3-C4 propriospinal neuronal system is unique since much of its organization and function is well known [20]. The C3–C4 PNs can mediate disynaptic excitation or inhibition from the cortico-, rubro-, reticulo-, and tectospinal tracts to forelimb motoneurons as shown schematically in Figure 2. In addition to the projection to forelimb motoneurons [39], [40], the C3–C4 PNs have a direct projection to neurons in the lateral reticular nucleus [41], [42], which is a major mossy fiber input to the cerebellum from the spinal cord. By way of this efferent copy, cerebellum can quickly correct for errors just prior to movement onset and during the movement [42]. The C3–C4 PNs are controlled by feedforward inhibition from all the descending systems described above and by feedback inhibition from muscle and cutaneous afferents in the forelimb [43]–[45].

Based on these neurophysiological connections of PN [20], [25], a model of the C3–C4 PN system was constructed as shown in Figure 2. It was assumed that an 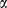-dynamic (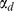) command of double tremor frequency was the direct driving signal for antagonistic muscles [7], and a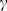-dynamic (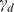) command was used to steer activations of antagonistic muscles for movement acceleration and deceleration [46]. The PN network seemed appropriate for such a task of alternating control of antagonistic muscle activations in rhythmic movements [26]. Combining these lines of physiological evidence, the following equations described the integration of cortical commands at the PNs, which computes PN outputs to the motoneuronal pools of antagonistic muscles:
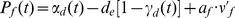
(1a)

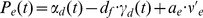
(1b)

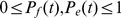
(1c)where 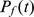, 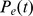 are the PN outputs to the 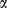-MNs of flexor and extensor muscles; 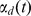 is the descending cortical commands of double tremor frequency; 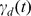 is the descending cortical 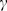 command of single tremor frequency; 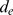 and 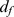 are the inhibition gains of descending 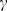 command; 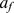 and 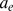 are the feedback gains of *Ia* afferent to PN; 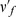 and 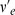 are proportional to *Ia* afferent discharge frequencies of spindles arising from flexor and extensor muscles. Subscripts f and e denote variables pertaining to flexor and extensor respectively. Note that 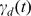 and its mirror signal, (1 - 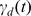), inhibit the PNs of flexor and extensor muscles in a reciprocal manner.

### Cortical Commands

The central commands originated from primary motor cortex (M1) may activate the 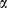-MNs in the motoneuron pool at anterior horn of spinal cord through two major corticospinal pathways in the CS-VA model: the mono-synaptic pathway and the multi-synaptic pathway (Figure 1). The multi-synaptic pathways from M1 to 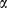-MN pool has been discovered in cat, monkey and human, while the mono-synaptic direct pathway appears to be unique to higher primates [14], [16]. The corticospinal pathways transmit and/or process specific groups of 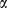 and 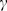 motor commands. According to the notion of dual control [29], [47], [48], a set of static commands, alpha static (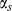) and gamma static (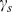) commands, were specified to maintain posture of the VA [36]. In addition, a set of dynamic commands, alpha dynamic (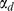) and gamma dynamic (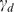), were assumed to produce tremor behaviors in the VA.

Based on the findings of Taylor et al. [46], [49], [50], gamma dynamic (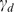) activity was correlated to an alternating control pattern during locomotion in decerebrated cat, which was similar to that in the Parkinsonian resting tremor in PD patients and in mimicking Parkinsonian resting tremor in healthy subjects. Thus, it was assumed that the 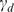 command was associated with the cortical oscillatory signal at single tremor frequency in the Parkinsonian tremor movements. In a correlation study [7], [8], [11], Timmermann and colleagues found that the cortical oscillatory activity at double tremor frequency had a stronger correlation to peripheral muscle EMG than that of single tremor frequency. This implied that the cortical signal of double tremor frequency would have a more direct influence on muscle activation during tremor movement. Therefore, in this model, the cortical signal of double tremor frequency was assumed to be associated with 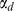 command that activated 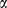-MNs of muscles via the PN network.

**Figure 3:**
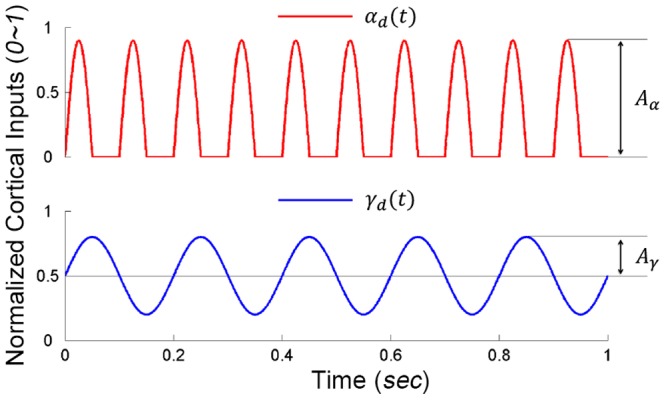
The cortical commands of alpha dynamic 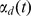 and gamma dynamic 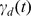 during 1 *sec*. 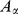
 denotes the amplitude of alpha command and 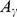 denotes the amplitude of gamma command with respect to a constant bias of 0.5.

In simulation experiments, we chose sinusoidal signals of single and double tremor frequencies to approximate the 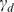 and 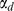 descending commands. They were input signals to the PN network or the motoneurons directly. The frequency range of the 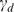 command was from 3∼8 *Hz*, and that of the motor command of 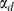 was 6∼16 *Hz*. The range of 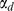 and 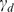 inputs were normalized to 0∼1. There was no phase shift between 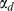 and 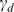. The central oscillatory inputs drove each pair of antagonistic muscles independently. The 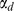 and 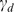 were described as follows:
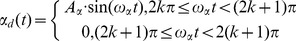
(2a)

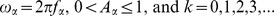
and
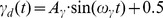
(2b)

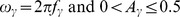
in which 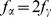, and the 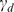 command was offset by a bias of 0.5 (see Figure 3). This bias would provide a static inhibition to the PN in resting state [20]. The nominal single tremor frequency used in this study was 5 *Hz*, which appeared to be the median tremor frequency we observed in PD patients [24].

### Simulation Experiments

Simulation experiments were performed with the protocol established in previous simulation studies [30], [38]. In all simulations, a set of static commands (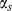 and 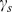) was specified to maintain the VA at a specific posture (see Table 1); and a set of dynamic commands (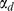 and 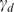) drove the model to elicit tremor behaviors with and without PN network.

#### Simulations without PN Network

The first set of simulation experiments was performed using the CS-VA model without the PN network. In this case, postural commands, (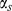 and 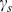), were impinged upon the 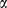-MN pool directly. The cortical command of double tremor frequency 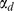 was passed down to the 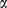-MN pool directly via the mono-synaptic corticospinal pathway, and the cortical command of single tremor frequency 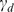 activated the 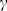-MN pool via the mono-synaptic corticospinal pathway. In this case, the 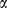-MN pool was still modulated by spinal reflexes of proprioceptive afferents of *Ia* and *Ib* origins, as well as recurrent inhibition [29], [30]. The gains of spinal reflexes were chosen within their stable ranges [30], and the values were given in Table 1. In this set of simulations, the amplitudes of 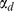 and 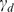 were varied from 0 to 1 and 0 to 0.5 respectively.

#### Simulations with PN Network

In the second set of simulation experiments, the PN network model of (Figure 2) was embedded in the corticomuscular coupling to mediated the cortical commands, (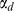 and 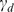), to the 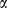-MN pool of muscles. Thus, additional corticospinal processing of descending commands was carried out in the PN network based on eqs. (1a) & (1b). This process played the pivotal role to partition the descending cortical commands into alternating muscle activation patterns between flexor and extensor muscles. However, the set of postural commands, (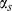 and 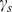), was still acted upon the 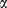 and 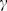 motoneuron pools directly. The activation level of 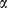-MN pool was modulated by spinal reflexes of proprioceptive afferents as with the other case [29], [30].

With PN network embedded in the corticomuscular model, the third set of simulation was then performed, in which the single tremor frequency of cortical commands was varied from 3 to 8 *Hz*, and double tremor frequency from 6 to 16 *Hz*. The objective of this set of simulations was to examine how cortical oscillations may be attenuated by peripheral damping in the neuromuscular system.

#### Analysis of Simulation Results

Simulated muscle inputs (U) and joint movements were analyzed off-line in MATLAB. Joint kinematic data were filtered by low-pass Butterworth filter with zero-phase shift, and cut off frequency of 20 *Hz* to eliminate sampling noise. The frequency spectra of joint kinematics and muscle activations were computed by MATLAB FFT algorithm. The amplitude of simulated tremor movements was evaluated with the averaged range of joint movement calculated for each tremor cycle.

Time delay and phase shift between the bursts of antagonist muscle inputs (U) were evaluated. A publically available MATLAB code (findpeaks.m, [51]) for peak detection was employed to locate the positive peaks in noisy signals of muscle neural input U. After locating the peaks of muscle bursts, the amplitudes and the peak times of bursts were then calculated. The time delay and phase shift between bursts of antagonist inputs were obtained as the difference of peak times. The time delay and phase shift of EMG bursts of antagonist muscles in PD patients were calculated similarly. The amplitude of bursts of muscle inputs in the third set of simulations was evaluated using this method.

### Hypothesis Testing

Two hypotheses were examined in this study with respect to the functional role of the propriospinal neuronal network in transmitting Parkinsonian tremor. Hypothesis I: the cortical oscillatory signals of Parkinsonian tremor are transmitted via the mono-synaptic corticospinal pathway without the PN network. In this case, the motoneurons of muscle are directly coupled to cortical motor outputs. Hypothesis II: the cortical oscillatory signals of Parkinsonian tremor are transmitted by way of propriospinal neuronal (PN) multi-synaptic corticospinal pathway. In this scenario, the PN network is involved in processing the descending cortical oscillatory signals.

Test of hypotheses was performed by comparing the simulated behaviors of the CS-VA model with and without PN network to those observed in PD patients. Decision of accepting or rejecting a hypothesis was based on the ability of the model to reproduce the signature features of kinematic tremor and EMG signals of PD patients qualitatively and/or quantitatively. The test criteria included (1) the occurrence of single tremor frequency peak in the spectra of simulated muscle inputs and joint movements, and (2) the occurrence of alternating activation pattern of antagonistic muscles, i.e. there was a significant phase shift between the simulated inputs to antagonistic muscles.

## Results

### Kinematic and EMG Characteristics of PD Tremor

Tremor and/or alternating burst EMGs in flexor and extensor muscles were observed in all seven PD patients during posture maintenance in the horizontal plane. The frequencies of tremor and EMGs of these PD patients ranged from 3.8 to 5.4 *Hz* with an average of 4.6 *Hz* [24]. The amplitude of oscillation was generally larger at the distal joint, particularly at the hand, than that at the proximal joint. The signature pattern of antagonist activation was the stereotyped alternating bursts in the flexor and extensor EMGs at all joints with the same frequency of tremor. Such a signature pattern was also evident in muscle EMGs, whose joint did not display an obvious tremor in some PD patients. Thus, the signature pattern of antagonist EMGs was a more reliable marker of tremor symptom in PD patients.

**Figure 4:**
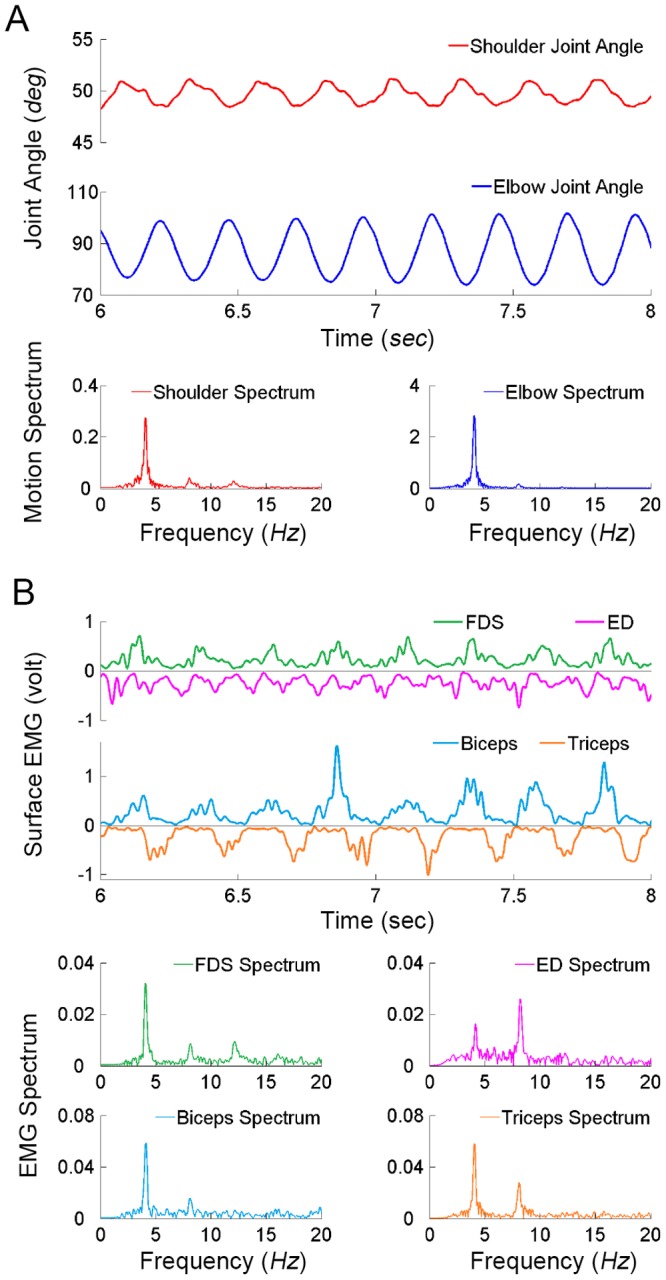
A segment of the kinematic data of 2-dominant PD patient is shown in (A). The shoulder and elbow angles of the right arm are presented respectively. Both elbow and shoulder show the oscillatory movements. The movement range of elbow is larger than shoulder. There is the same peak at around 4.11 *Hz* (the tremor frequency) in the frequency domain of shoulder and elbow. The processed EMG data in this 2 s segment of Biceps, Triceps, FDS (Flexor Digitorum Superficialis) and ED (Extensor Digitorum) are shown in (**B**). Both these two pairs of antagonist muscles show the alternating patterns of muscular activities. There are the single tremor frequency and the double frequency components in those muscular EMGs.

A segment of the kinematic and EMG data from the right arm of a tremor-dominated PD patient was shown in Figure 4. This set of data was collected with the shoulder joint maintained at about 50^0^ and elbow joint at about 85^0^. In Figure 4A, both shoulder and elbow joints showed oscillatory movements at the same tremor frequency. The amplitude of elbow oscillation was greater than that of the shoulder. FFT spectrum of the joint oscillations displayed a peak at about 4.11 *Hz* in both the shoulder and elbow. Showing in Figure 4B were the filtered EMG data of Biceps, Triceps, FDS (flexor digitorum superficialis), and ED (extensor digitorum). The alternating burst pattern of EMGs was clearly displayed in each pair of antagonist muscles. FFT spectra of all muscles presented a prominent single frequency component of tremor and declining harmonic components of tremor frequency. These results agreed with the features of resting tremor observed in PD patients [1], [52]–[54]. Average time delay and phase shift from Biceps to Triceps in a tremor dominant PD patient was 98.89±12.34 *msec* and 146.21±18.25 *deg*, from FDS to ED was 121.98±15.48 *msec* and 180.35±22.89 *deg* (Table 2). These experimental data were used as the test templates for simulated tremor behaviors.

**Table 2:** Calculated Phase Shifts Of Agonists To Antagonists From Simulated And Recorded Pd Tremor.

	Simulation Results of Muscle Activation*					Empirical Results of sEMG**		
		Without PN^1^		With PN				
Joints	Muscles	Time Delay (*msec*)	Phase Shift (*deg*)	Time Delay (*msec*)	Phase Shift (*deg*)	Muscles	Time Delay (*msec*)	Phase Shift (*deg*)
**Shoulder**	**PC** 2 **/DP** 3	−0.18±0.11	−0.65±0.41	100.05±1.96	180.10±3.52			
**Bi-articular**	**Bsh** 4 **/Tlh** 5	−0.29±0.15	−1.05±0.54	100.14±1.93	180.24±3.47	**Biceps/Triceps**	98.89±12.34	146.21±18.25
**Elbow**	**BS** 6 **/Tlt** 7	0.10±0.10	0.35±0.35	99.97±1.65	179.94±2.98			
**Digitus**						**FDS** 8 **/ED** 9	121.98±15.48	180.35±22.89
**Average**		**−0.13**±**0.20**	**−0.45**±**0.72**	**100.05**±**0. 08**	**180.09**±**0.15**		**110.44**±**16.33**	**163.28**±**24.14**

* Simulated Muscle Activation Frequency 5.00 Hz.

** Recorded Surface EMG Frequency 4.11 Hz.

1 Propriospinal Neurons.

2 Pectoralis major Clavicle portion.

3 Deltoid Posterior.

4 Biceps short head.

5 Triceps long head.

6 Brachialis.

7 Triceps lateral head.

8 Flexor Digitorum Superficialis.

9 Extensor Digitorum.

### Simulations without PN Network

**Figure 5:**
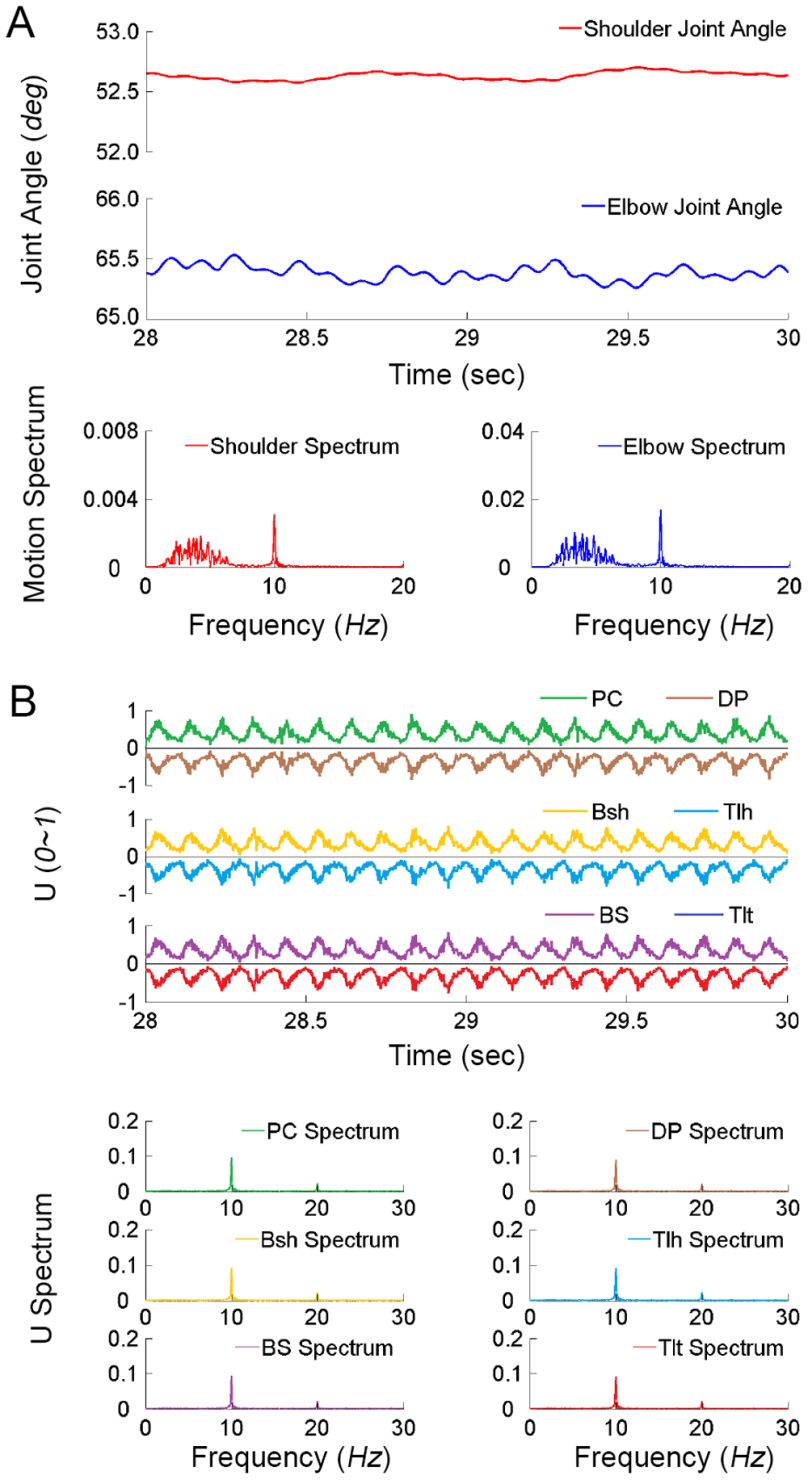
Result of a simulation without PN network is presented with 5/10 *Hz* as the representative pair of single/double tremor frequencies. (**A**) The joint movements of the virtual arm are presented. There are small fluctuations of 10 *Hz* both in shoulder and elbow joints. The spectrums of joint movements were calculated with a data window between 20 s∼30 s of simulation. There was no peak at 5 *Hz* in the amplitude of spectrum of both shoulder and elbow joints. In (**B**), the direct excitations (U) of all pairs of antagonistic muscles show a co-contraction pattern, and no alternating activations of antagonist muscles are evident. The neural inputs of muscles (U) show a peak at 10 *Hz* and its harmonic components in frequency spectrum.

**Figure 6:**
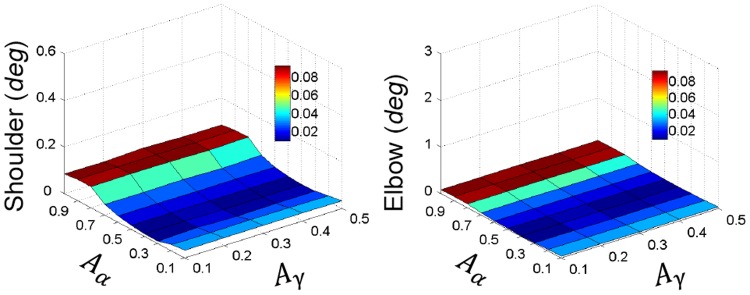
Results of the tremor amplitude of shoulder and elbow with varying 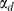 and 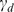 obtained without PN network are shown in 3-D maps as a function of cortical oscillation amplitudes of 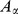 and 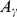. It is clear that for large ranges of cortical oscillations, no peripheral tremor at either 5 *Hz* or 10 *Hz* were evident, except for high 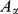 values above 0.7, where the small oscillation elicited was of double tremor frequency of 10 *Hz*. This may be due to direct pass of 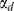
command onto motoneuron pools that cause co-contraction of antagonistic muscles.

Results from the first set of simulations using the CS-VA model were summarized in Figure 5, in which cortical commands were directly coupled to the motoneuron pools of muscles without the PN network. The single and double and tremor frequencies of the cortical commands, 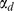 and 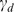, were 5 *Hz* and 10 *Hz* respectively. In general, the inputs of each pair of antagonistic muscles (U) showed an in-phase co-contracting bursts, but not the alternating pattern as seen in PD patients (Figure 4B). The dominant contents of frequency in all muscle inputs (U) were 10 *Hz* with little or no 5 *Hz* oscillations. The joints of the virtual arm did not show obvious tremor behaviors at 5 *Hz*. The single tremor frequency component of cortical commands was not translated in the muscle activities and joint tremors at all, because the 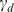 command activated muscle spindles only. In the full range of amplitudes of both cortical commands, the CS-VA model without the PN network did not produce PD-like tremor behaviors in both shoulder and elbow joints, as shown in Figure 6. What was consistent in the simulation without the PN network was a high level of in-phase co-contraction of antagonistic muscles at the double tremor frequency. This result was contradictory to the signature feature of alternating bursts in the flexor and extensor muscles observed in PD patients in Figure 3 and in literature [1], [52]–[54].

The flexor and extensor muscles showed almost a zero time delay and a zero phase shift in their neural inputs (U) (Table 2). This again indicated a co-contraction burst pattern of flexor and extensor muscles, which was not a favorable condition to generate rhythmic movement at joints. Based on these results, the hypothesis I was rejected as a plausible mechanism of corticomuscular transmission of tremor commands.

### Simulations with PN Network

**Figure 7:**
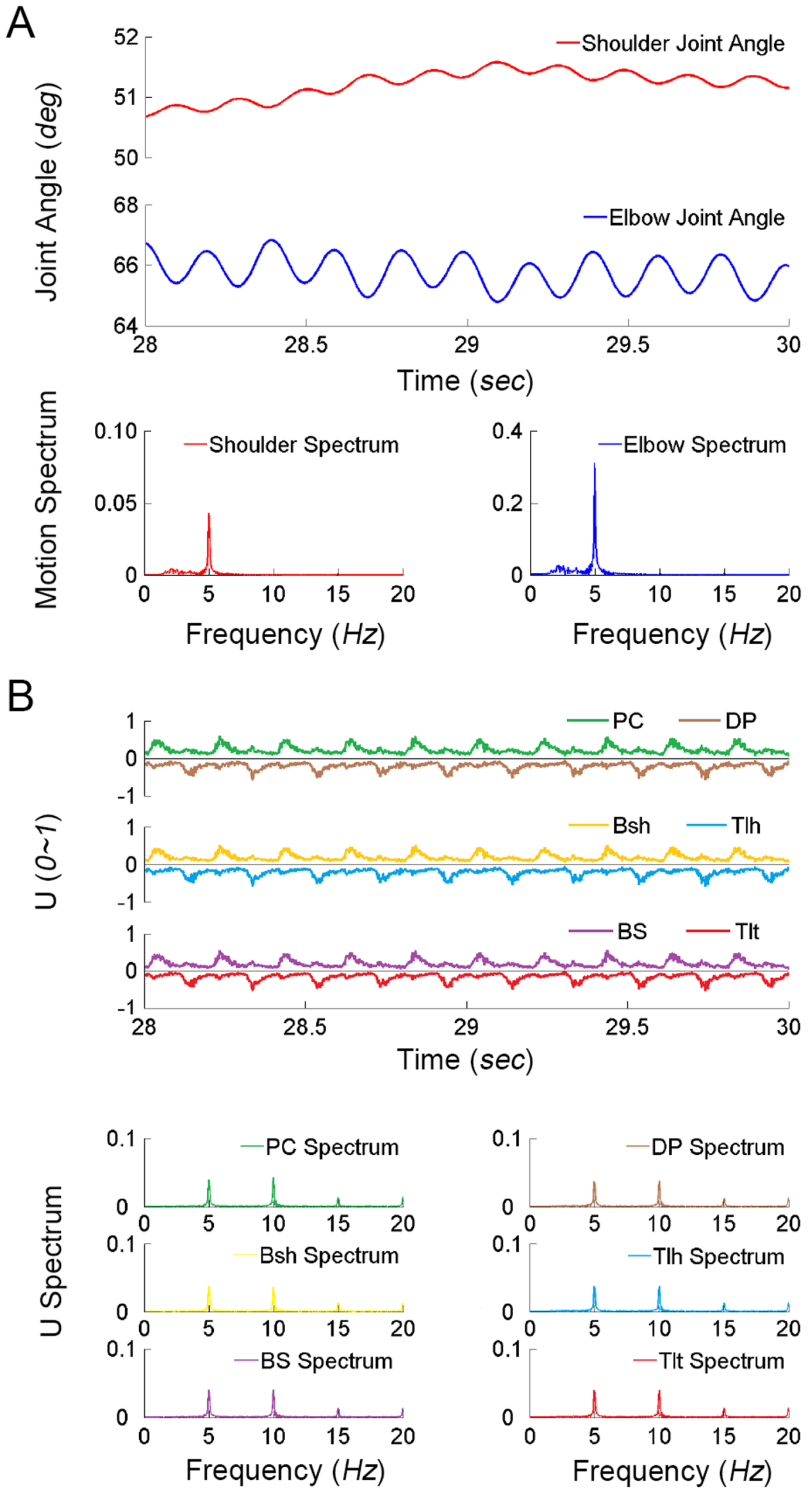
Results of simulation with PN network are presented. (**A**) The virtual arm reveals obvious oscillatory movements of 5 *Hz*. The elbow joint displays a larger oscillation at 5 *Hz* than the shoulder joint. (**B**) The outputs of 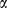 motoneuron pool of antagonistic muscles (U) show an alternating pattern of activation. The neural inputs of three pairs of antagonistic muscles (U) show a peak frequency at 5 *Hz*, as well as declining harmonic components in the frequency spectrum. There is a phase shift between activations of flexor and extensor muscles. The time delay and phase shift are calculated in Table 2.

The results from second set of simulations were presented in Figure 7, in which the cortical commands were relayed to the MNs via the PN network. In this case, the cortical commands were processed in the PN network to produce inputs to the MN pools of muscles. The muscle inputs (U) to the VA model displayed an alternating burst pattern in each pair of antagonistic muscles, which directly contributed to the alternating activation of antagonist muscles. The dominant contents of frequency in U of all muscles were 5 *Hz*, but there were also declining harmonic components at 10 *Hz* and 15 *Hz* in the muscle inputs, which were similarly observed in EMGs of PD patients. The virtual arm displayed tremor like behavior of 5 *Hz* primarily at the elbow joint accompanied by a smaller oscillation of 5 *Hz* at the shoulder joint. The higher frequency components in muscle inputs (U) were filtered out by the low-pass filter effect of neuromuscular dynamics (see section 4 in the following). The simulated joint oscillations captured the general characteristics of pathologic Parkinsonian resting tremor and the signature feature of PD patients presented in Figure 4.

**Figure 8:**
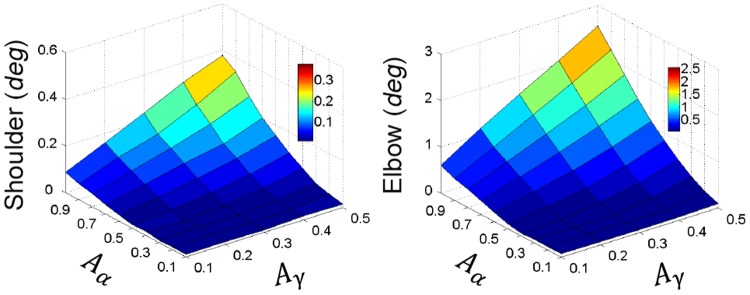
Results of the oscillatory amplitude of shoulder and elbow with varying 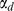 and 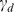 obtained with PN network are shown in 3-D maps as a function of cortical oscillation amplitudes of 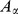 and 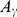. Oscillatory amplitudes of shoulder and elbow joints are both increased with amplitude of central oscillations. The tremor amplitude of elbow is varied more significantly than that of shoulder. It appears that 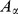 has a stronger influence on peripheral tremor because at low 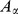, no peripheral tremor can be initiated by 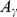. And at high 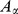, the amplitudes of peripheral tremor climbed rapidly. This suggests that 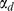 is the direct driving command for muscles.

The simulated tremor behaviors within the full range of cortical commands of 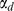 and 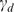 were summarized in Figure 8. The results indicated that Parkinsonian tremor could be reliably reproduced by the CS-VA model with the PN network embedded in the corticomuscular transmission. In general, the oscillation amplitude of both shoulder and elbow joints were proportionally increased with the increase in the amplitude of 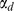, as well as 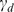 at high levels of 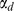. It was shown that 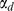 had a stronger influence to tremor activities in the periphery, which was consistent with the finding that double tremor frequency oscillation in the motor cortex (M1) was more directly correlated to peripheral muscle EMGs [11].

The calculated time delay between neural inputs of flexor and extensor muscles (Table 2) was about 0.1 *sec* on the average, and the phase shift was about 180 *deg*. Thus, the PN network was able to create a phase shift between flexor and extensor activations that were favorable to produce oscillatory movements.

### The Effects of Neuromuscular Damping

**Figure 9:**
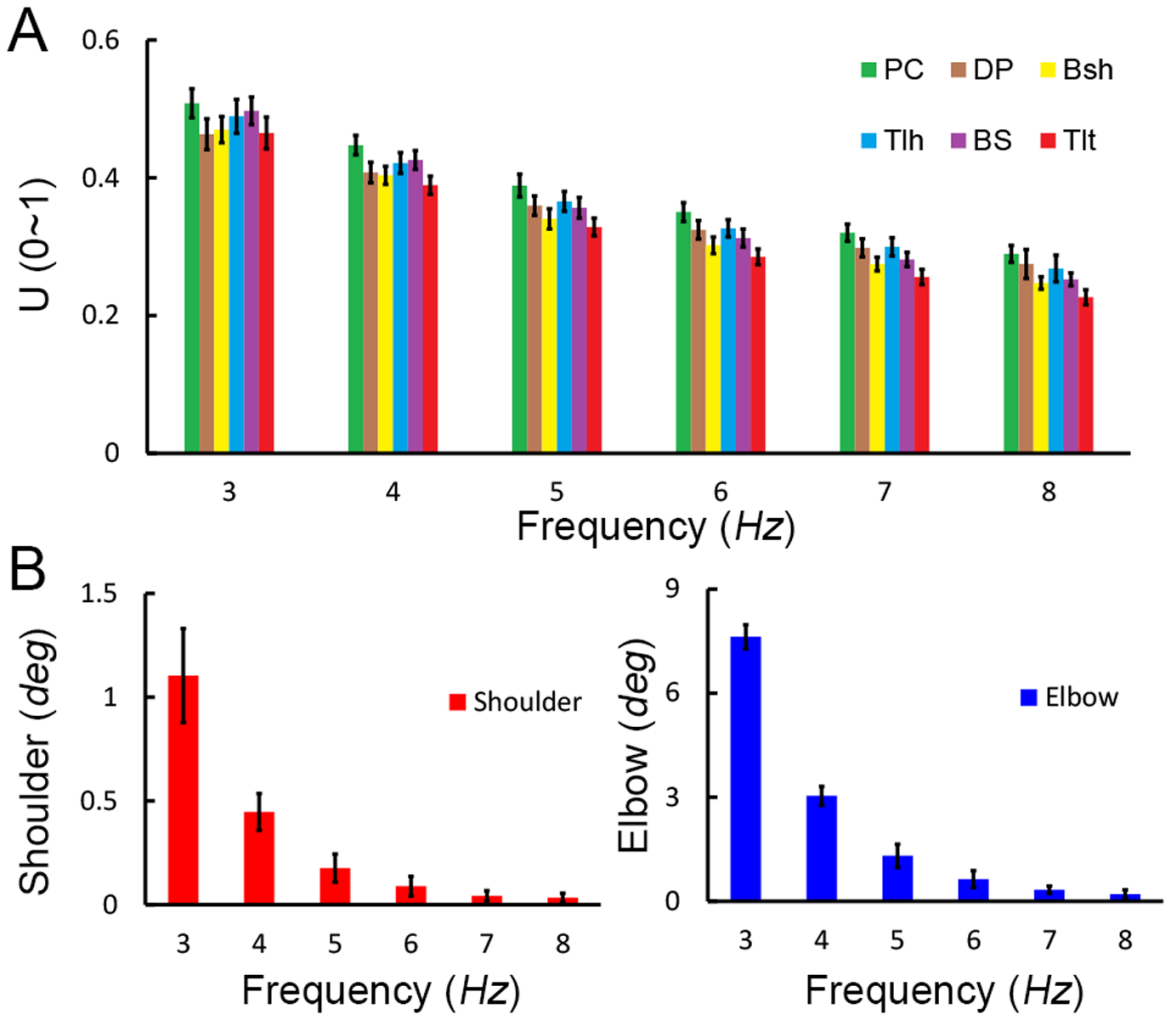
Results of frequency dependent damping effects on tremor by neuromuscular dynamics were obtained with cortical inputs at a high level (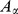 = 0.9 And 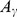 = 0.3) across the range of tremor frequency (3∼8 *Hz*). Different colors represent variables of different muscles. (A) The amplitude of muscle inputs (U) was presented, which showed a frequency dependent attenuation. This damping effect was mainly due to the excitation dynamics of motoneuron pools. This built-in excitation dynamics was embedded into the spinal reflexes of the VA model. (B) The overall frequency dependent damping effects on shoulder and elbow tremors were shown. The total damping effect of neuromuscular dynamics included the contributions of excitation of motoneuron pools, the muscle activation-contraction dynamics, and the inertia and viscosity of the limb. It was shown that the damping effect was greater with higher tremor frequency. In particular, at the tremor frequency of 8 *Hz*, the amplitude of tremor at both shoulder and elbow joints diminished to almost zero.

In the third set of simulation experiments, how Parkinsonian tremor of different frequencies may be attenuated by neuromuscular dynamics were evaluated, and the results were shown in Figure 9. In these simulations, the amplitude of tremor with frequency from 3 to 8 *Hz* was analyzed with constant amplitude of cortical oscillations. In Figure 9A, the amplitude of muscle inputs (U) displayed a varying degree of weakening at each frequency due to the excitation dynamics in the activation of motoneuron pool [29], [30], which was higher with higher frequency. However, the tremor was further dampened at the musculoskeletal joint as shown in Figure 9B, where joint oscillations of higher frequency were reduced more prominently than that of lower frequency. At the frequency of 8 *Hz*, the tremor amplitude diminished to almost zero. This decrease in tremor amplitude was due to the low-pass filter nature of neuromuscular dynamics [29], [30]. The total damping effect of neuromuscular dynamics included the contributions of excitation of motoneuron pools, the muscle activation-contraction dynamics, and the inertia and viscosity of the limb. This result agreed with experimental observations in literature, in which tremor activities of 3∼7 *Hz* were typically observed in PD patients [2], [3].

## Discussion

### Modeling of Corticomuscular Transmission of Tremor Signals

In this study, we address the question whether the PN network is involved in corticomuscular computation of antagonist muscle activations from cortical oscillatory commands in the generation of Parkinsonian tremor. Evidence accumulated from normal rhythmic movement studies [21], [22] and cortical MEG correlations with peripheral muscle EMGs [7], [8] and [11] suggests that there exists a spinal circuitry in the coupling from cortical oscillations to muscular activities. It is counterintuitive, however, that the cortical oscillation at double tremor frequency is found more strongly correlated to the peripheral muscular activity than that of single tremor frequency [7], [8] and [11]. The latter would be thought of a more straightforward signal to elicit tremor in the periphery for their similar frequency of oscillation. This experimental fact implies that the cortical signal of single tremor frequency might not be passed down to activate the alpha motoneuron pools of muscles. The alternative pathway the single tremor oscillations to influence peripheral activities would be through the gamma motoneurons innervating muscle spindles. Indeed, recording of 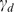 activities from locomotion in behaving animals [46] provided clues of an in-phase bursting of 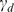 firing with movement speed, showing that the 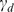 bursting frequency was the same as that of the rhythmic locomotion. Thus, in this study of PD tremor simulation, we assume that 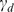 is associated with the cortical oscillation of single tremor frequency, while the cortical oscillation of double tremor frequency is passed down to activate the alpha motoneurons of muscles. The question addressed in this study is then whether the corticomuscular coupling is through the mono-synaptic corticospinal pathway or the multi-synaptic corticospinal pathway.

A bottom-up modeling and computational approach is taken in this study to elucidate this question combined with experimental observation of tremor kinematic and EMG behaviors in PD patients [24]. In particular, a model of the PN network based on its physiological connections [20], [25] has been constructed as shown in Figure 2, as well as in equations (eq.1). A previously validated virtual arm (VA) model with a spinal reflex (SR) circuitry [30] is extended to receive cortical descending inputs from M1 via either the mono-synaptic pathway or the multi-synaptic pathway with the PN network model (Figure 1). This corticospinal virtual arm (CS-VA) model embeds the realistic PN processing of cortical commands [20], [25], the regulation of spinal reflexes [29], [30], the natural recruitment and activation dynamics of muscle fibers [31], [35], and the accurate biomechanical actions of muscles at joints [34]. The CS-VA model can, thus, allow us to address issues that would be impossible to perform using invasive experimental procedures. The CS-VA model could also produce insights of pathological signals in motor cortex that can be corroborated with many clinical diagnostic methods, such as MEG, EMG and EEG. Furthermore, with the techniques of recording using micro-electrode arrays and probes [55], the abnormal neurophysiological events in the basal ganglia loop [9], [56]–[60] can be correlated with cortical signals of neural activities, which can then be examined in the CS-VA model to understand the pathophysiology of PD tremor. The model could be useful to understand the mechanism of deep brain stimulation (DBS) to treat PD symptoms [61], [62]. Nevertheless, the model is suitable only for simulation of PD tremor behaviors with cortical driving oscillations pertaining to PD markers.

Figure 9 illustrates clearly that the tremor oscillations are dampened at the periphery by the neuromuscular dynamics. The damping effects are more prominent for higher frequency tremor due to its low-pass filtering nature. The total damping effect of neuromuscular dynamics included the contributions of excitation of motoneuron pools, the muscle activation-contraction dynamics, and the inertia and viscosity of the limb. Acting together, these damping factors may have prevented tremor above 8 *Hz* to occur. Indeed, only tremors between 3–7 *Hz* are clinically observed in PD patients [2], [3]. In our experimental measurements, the tremor frequency of PD patients ranges from 3.8 to 5.4 *Hz*, which is well within the range of clinical PD tremor. Our model is able to make such a prediction because it contains realistic neuromuscular dynamics and accurate biomechanics of the limb [29]–[38]. This agreement with clinical observations in turn further verifies the validity of the CS-VA model developed in this study.

### The Role of PN in Transmitting PD Tremor

Simulation results indeed demonstrates that the CS-VA model with PN network can reliably reproduce the signature feature of tremor behaviors of PD patients, i.e. the alternating bursts of flexor and extensor muscle activities in the full range of central parameters of cortical commands and frequencies (Figures 4, 7, 8&9 and Table 2). The alternating bursts in the flexor and extensor muscles are favorable condition to generate oscillations in the musculoskeletal joints. The similar type of burst pattern is also displayed in voluntarily generated rhythmic locomotion movements [46] and in PD patients [24]. The simulated tremor amplitude is larger at the distal joint of the arm, and smaller at the proximal joint of the arm, such as the shoulder (Figure 7A), because of its larger inertia of movement. This is consistent to the characteristics observed in PD patients in Figure 4. The variation in tremor amplitude at different joint locations is, thus, due to the biomechanical properties at the joints, rather than any neurological difference in the cortical driving signals to muscles of different joints.

The results clearly illustrate that without the PN network, the tremor behaviors cannot be reproduced with the CS-VA model. In this case, a high level of co-contraction bursts persists in the antagonist muscles, which prevents the generation of tremor at joints. This observation alone is sufficient to reject the first hypothesis, and rule out the possibility that the cortical oscillatory signals of Parkinsonian subjects are transmitted via the mono-synaptic corticospinal pathway bypassing the PN network. This outcome also suggests that the spinal reflex circuits alone are not sufficient to partition the central oscillatory signals into alternating burst activities in the antagonistic muscles in the range of stable spinal reflex gains.

The results of simulation substantiate the second hypothesis that a corticomuscular computation is performed at the premotor spinal level in order to produce alternating bursting activities at the antagonist muscles. The PN network is shown to be able to perform the computation of the alternating activations of flexor and extensor muscles for a wide range of tremor behaviors. This function can be accredited to the mirror-gaiting inhibition of 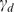 from motor cortex to the PN. The mirror-gating mechanism at the PN was implemented in eq. (1a) & (1b). The PNs of the flexor and extensor muscles were inhibited by cortical descending commands of 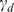 and (1 - 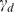), respectively. This would allow two consecutive pulses in the 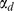 signal would be split into two single pulses with phase shift, each of which was channeled to one of the antagonistic muscles. Thus, the PN network essentially divides the 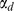 command of double tremor frequency into the alternating pattern of activation at single tremor frequency for the flexor and extensor, respectively. This computational function is evaluated in the full range of cortical commands of tremor generating conditions (Figures 8 & 9), thus, validating the role of PN network computation in tremor elicitation.

Basal ganglia circuitry has two outputs, one of which is fed back to cortex via thalamocortical pathway. This signal is relayed to spinal C3–C4 propriospinal neurons through primary motor cortex. The other output of basal ganglia is passed down to the brainstem and to the lumbar segment of spinal cord to control locomotion [63], [64]. Thus, the likely alternative spinal mechanism may exist in the lumbar segment of spinal cord, where the Central Pattern Generators (CPGs) of locomotion are located. The CPGs are themselves oscillators, which generates repetitive sequences of muscle activations to elicit locomotion [65]–[67]. However, in the case of PD, it would be possible that the CPGs are synchronized to give rise to alternating activation pattern of antagonistic muscles in the lower extremity, which would cause tremor activity. The CPG networks may work differently from the PN networks, and the mechanism of CPG initiated tremor needs to be further elucidated.

The effect of proprioceptive afferents on tremor in PD patients is largely unclear. However, the spinal reflex gains in PD patients did not seem to be drastically different from those of normal subjects [68]–[71], except for Ib reflex gain, which was found reduced in PD patients [19]. It was shown that increased Ib inhibition gain would lower the amplitude of tremor [72]. The more complex situation is the extensive connections of group II afferents in the spinal circuitry via interneurons [69], [73], which form the long-loop reflexes. Their multi-synaptic interactions make it difficult to predict their effects on Parkinsonian tremor [19]. Finally, PNs receive excitatory and inhibitory innervations from both cutaneous and proprioceptive afferents [20]. Their effects on tremor activity would depend on the relative potency of excitation and inhibition to the PNs. A better understanding of the proprioceptive influence on tremor requires elucidation of pathophysiology of PD with respect to spinal reflex circuitry [19].

### The Nature of Cortical Oscillations

A relevant issue regarding the organization of cortical oscillations of PD tremor can also be implicated using the CS-VA model of Figure 1. There has been suggestion in the literature that there are more than one cortical oscillation modules, each of which may affect the muscles at different limb of the body [51], [74]–[76]. Our observation in PD patients indeed reveals a common frequency of tremor and muscle EMGs at shoulder and elbow joints in the same arm. It implies that a single module of cortical oscillations of single and double frequencies is passed down to the spinal motoneuron pools to excite all muscles of one limb in parallel. This idea is verified using simulation here, in which a single set of cortical commands is used to drive three pairs of antagonist muscles in parallel, each controlling the shoulder and/or elbow joints, respectively. The simulated tremor behaviors and muscle inputs display the similar frequency to that of cortical oscillations, as is observed in the tremor behaviors of PD patients [24]. The results appear to substantiate the proposal of [74] and [75] that a single module (or source) of central oscillations affects the tremor of one limb of the body.

In this study, a dual set of cortical command signals are implemented for postural maintenance (static commands, 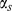 and 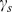) and oscillation elicitation (dynamic commands, 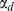 and 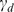) respectively. The mono-synaptic corticospinal pathway transmits static postural commands, and the multi-synaptic corticospinal pathway performs further processing of the dynamic tremor commands. The separate corticospinal neural pathways for transmitting postural and tremor commands imply that it is possible that there exist separate modules in the motor system for control of posture and movement tasks. This is in line with the previous notion of dual control framework for posture and movement [29], [47], [48]. In a more extensive set of experiments [24], the PD subjects performed both posture and reaching tasks in a horizontal plane. The PD subjects were able to maintain different postures with superimposing tremor activities, but the reaching movement was performed significantly slower than normal. Interestingly, the tremor activity was suppressed during reaching tasks. This implies that the posture module in PD patients appears to be intact, while the movement module is contaminated by involuntary oscillatory signals, which are passed down to periphery to cause tremor. Deep brain stimulation (DBS) of the subthalamic nucleus (STN) appears to mask the involuntary oscillatory signals in the movement module, thus, stopping the tremor symptom [61], [62].

## Conclusion

A realistic model of the corticospinal virtual muscle (CS-VA) system is applied to replicate tremor behaviors in PD patients. The agreement of simulated tremor behaviors with those observed in PD patients supports the hypothesis that cortical tremor commands are transmitted downstream to the motoneuron pools of muscles via the propriospinal neuron (PN) pathway. The PN network in the C3–C4 levels is revealed to play the pivotal role in computing the alternating burst pattern of flexor and extensor EMGs by a mirror-gaiting mechanism. This computational role is further evaluated in the full range of tremor generating conditions in comparison with behaviors observed in PD patients. The CS-VA model also demonstrates the frequency dependent damping effect on tremor activities by neuromuscular dynamics. The high frequency damping may have prevented tremor above 8 *Hz* to occur in PD patients. The agreement with clinical observations in turn substantiates the validity of results of model predictions.
